# Causes and risk factors for singleton stillbirth in Japan: Analysis of a nationwide perinatal database, 2013–2014

**DOI:** 10.1038/s41598-018-22546-9

**Published:** 2018-03-07

**Authors:** Rei Haruyama, Stuart Gilmour, Erika Ota, Sarah K. Abe, Md. Mizanur Rahman, Shuhei Nomura, Naoyuki Miyasaka, Kenji Shibuya

**Affiliations:** 1 Department of Global Health Policy, Graduate School of Medicine, The University of Tokyo, Tokyo, Japan; 2 Department of Global Network and Partnership, Bureau of International Medical Cooperation, National Center for Global Health and Medicine, Tokyo, Japan; 3 Global Health Nursing, Graduate School of Nursing Science, St. Luke’s International University, Tokyo, Japan; 4 Institute for Global Health Policy Research, Bureau of International Medical Cooperation, National Center for Global Health and Medicine, Tokyo, Japan; 5 Comprehensive Reproductive Medicine, Graduate School, Tokyo Medical and Dental University, Tokyo, Japan

## Abstract

Over 80% of perinatal mortality in Japan is due to stillbirths after 22 weeks of gestation, with one in 300 families experiencing fetal loss every year. This study aimed to assess causes and risk factors for singleton stillbirth in Japan. A retrospective cross-sectional study was conducted using the Japan Society of Obstetrics and Gynecology Perinatal Database from January 2013 to December 2014. A total of 379,211 births including 2,133 stillbirths were analyzed. Causes of death were classified into eight categories. A multi-level Poisson regression model was used to assess the relationship between stillbirth and key covariates. Causes of death were unknown in 25–40% of stillbirths across gestational age. Placental abnormality accounted for the largest proportion of known causes, followed by umbilical cord abnormality. Stillbirth risk was increased among small-for-gestational-age infants (adjusted relative risk [ARR]: 3.78, 95% confidence interval [CI]: 3.31–4.32) and nulliparous women (ARR: 1.19, 95% CI: 1.05–1.35). Maternal underweight, pregnancy-induced hypertension and oligohydramnios showed a protective effect. Our finding suggests that stillbirths occurring among women with known complications are likely already being prevented. Further reduction in stillbirths must target small-sized fetuses and nulliparous women. Improved recording of the causal pathways of stillbirths is also needed.

## Introduction

Globally, 2.6 million third trimester stillbirths are estimated to occur annually, with a rate of 18.4 per 1000 births in 2015^1^. Although 98% occur in low- and middle-income countries^1^, stillbirth accounts for the largest proportion of perinatal deaths in high income countries^2,3^. In Japan, the perinatal mortality rate has substantially dropped over the past 30 years to become one of the lowest in the world, due to advances in health technology such as intrapartum fetal monitoring as well as improvements in the perinatal care system^3–5^. However, more than 80% of perinatal mortality is due to stillbirths after 22 weeks of gestation, with a rate of 3.0 per 1000 births^6,7^. This gives an annual loss of 3,000–3,500 fetuses, higher than all deaths in children under five years of age. Nevertheless, epidemiological studies of stillbirth are scarce in Japan. Postmortem investigation for identifying probable cause of death and counseling for families experiencing stillbirths also appears to be insufficient and varies among facilities compared to those offered after an infant death^8^.

One available study on stillbirth, using the Japan Society of Obstetrics and Gynecology (JSOG) Perinatal Database from 2001 to 2004, reported the cause of death was unknown in 25.0% of stillbirths, and placental abruption (17.8%), congenital malformation (17.0%), and umbilical cord abnormality (16.1%) accounted for most stillbirths among the known causes^9^. However, this finding is difficult to interpret, as the results of singleton and multiple pregnancies are combined. It is also not clear at which gestational age these conditions are most frequently observed and attributed as cause of death. To date, no studies have examined risk factors for stillbirth in Japan. A meta-analysis of studies from 13 high-income western countries showed that maternal overweight and obesity, advanced age, and smoking are the highest ranking modifiable risk factors for stillbirth^10^. However, these findings may not be generalizable to a population with different health risks such as a much lower mean body mass index (BMI), as in Japan^11^.

To provide better clinical knowledge and help formulate strategies to reduce preventable fetal loss in the country, this study aimed to examine causes and risk factors for singleton stillbirth in Japan.

## Methods

### Data source

This study used the JSOG Perinatal Database from January 2013 to December 2014. The details of the database have been described elsewhere^12–14^. In brief, the database is a nationwide registry that contains clinical information for all births after 22 weeks of gestation at registered participating obstetric facilities, of which 75% are secondary and tertiary centers. At each facility, clinical data for women who gave live births or stillbirths are entered into the database by doctors, midwives, or trained data clerks. Anonymized data are sent to the JSOG Perinatal Committee and annual reports are made publicly available online^15^. Table 1 compares the number of total births, stillbirths and obstetric facilities between the national data and JSOG Perinatal Database in 2013 and 2014^6,16^. Over the study period, a total of 406,287 births including 2,514 stillbirths were recorded in the database, corresponding to 19.9% of all births and 40.9% of stillbirths in Japan. This represents a stillbirth rate of 6.2 per 1000 births (95% CI: 6.1–6.3), higher than the national rate of 3.0 per 1000 births.

**Table 1:** Comparison between the national data and Japan Society of Obstetrics and Gynecology Perinatal Database.

Year	National data^a^			JSOG Perinatal Database		
	Total births	Stillbirths^b^	Facilities^c^	Total births (% registered)	Stillbirths (% registered)	Facilities (% registered)
2013	1,032,926	3, 110	—	186, 235 (18.0)	1, 172 (37.7)	299
2014	1,006,578	3, 038	2,363	220, 052 (21.9)	1, 342 (44.2)	355 (15.0)
Total	2,039,504	6, 148		406, 287 (19.9)	2, 514 (40.9)	

^a^Source: Ministry of Health, Labour and Welfare.

^b^Spontaneous stillbirths after 22 weeks of gestation.

^c^Facilities handling deliveries (1,055 hospitals and 1,308 clinics).

JSOG: Japan Society of Obstetrics and Gynecology.

### Sample selection

**Figure 1:**
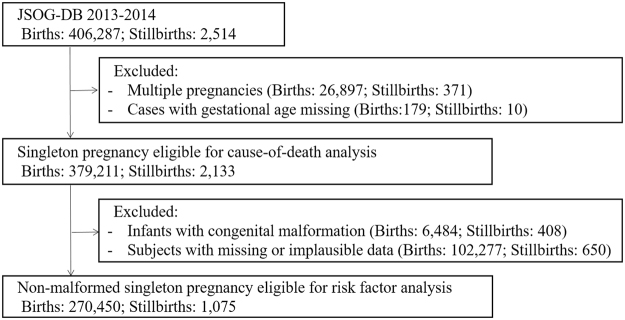
Flow diagram of the study sample. The Japan Society of Obstetrics and Gynecology Perinatal Database from January 2013 to December 2014 was used in this study. After excluding multiple pregnancies and cases with missing gestational age, cause of death was analyzed for 2,133 singleton stillbirths. For risk factor analysis, infants with congenital malformation and subjects with missing or implausible data were excluded, giving 270,450 singleton births and 1,075 stillbirths eligible for analysis.

Figure 1 shows the study design. After excluding multiple pregnancies and cases with missing gestational age, cause of death was analyzed for 2,133 singleton stillbirths (84.8% of initial data). For risk factor analysis, infants with any kind of congenital malformation (i.e., those who died of congenital malformation, died of other causes but had congenital malformation, and did not die but had congenital malformation) were further excluded to remove the effect of potentially unavoidable stillbirth related to these conditions. Subjects were also excluded when any of the following data were missing or implausible: maternal age, parity, pre-pregnancy weight, height, smoking status, infant sex and birth weight. Maternal weight below 30 kg or over 150 kg, and height below 130 cm or over 200 cm were considered implausible. A total of 270,450 singleton births (66.6% of initial data) and 1,075 stillbirths (42.8% of initial data) were eligible for analysis. There were statistically significant differences in gestational age distribution, maternal age, parity, pre-pregnancy BMI and smoking status between included and excluded subjects (see Supplementary Table S1). However, the differences were clinically negligible.

### Outcome variable

In Japan, stillbirth is defined as intrauterine fetal death after 22 weeks of gestation, after which termination of pregnancy is essentially prohibited^17^. In the JSOG Perinatal Database, timing of death (stillbirth, early neonatal, or late neonatal), cause of death, and autopsy and placental pathology results (if performed) are recorded for perinatal deaths. The single most probable cause of death is recorded using the 17 categories of the JSOG clinical death classification system (see Supplementary Table S2). Optionally, more detailed conditions are reported in the free-text column. When an infant’s outcome was recorded as death but information on its timing was missing, the case was reviewed for details such as Apgar scores, resuscitation attempt, and comments in the free-text column, and counted as stillbirth if intrauterine death was confirmed.

Causes of death were reclassified and examined in accordance with level 1 categories of the Cause of Death and Associated Conditions (CODAC) classification system^2,18^. In the CODAC system, the primary cause of death is entered at three levels. At level 1, one major contributor is chosen from eight categories (infection, intrapartum, congenital anomaly, fetal, cord, placenta, maternal, and unknown) and more detailed conditions are chosen from sub-categories in levels 2 and 3. Using the level 1 categories of this system allowed for comparison with previous literature and minimized risk of misclassification from the JSOG clinical death classification system to CODAC. Stillbirths attributed to “low birth weight with other causes” were considered as deaths due to unspecified fetal condition.

### Explanatory variables

For risk factor analysis, maternal and fetal factors previously reported to be associated with stillbirth were analyzed^10,19–29^. The type of facility by level of service was also examined, because an association with adverse obstetric and perinatal outcomes has been reported in some studies, although not all^30,31^. Gestational age was determined from the last menstrual period or ultrasound measurements^32^. Pre-pregnancy BMI was calculated from the self-reported height and weight. BMI of 23 kg/m^2^ was used as the cut-off for normal weight on the basis of potential increase in various health risks for Asian populations with BMI over 23 kg/m^2^ ^33^. Oligohydramnios and polyhydramnios were determined by routine ultrasound measurement of amniotic fluid index or single deepest pocket^32^. An infant was considered small-for-gestational-age (SGA) or large-for-gestational-age (LGA) if the birth weight was below or above the 10th percentile of the Japanese optimal birth weight standard by gestational age^34^. The diagnosis of fetal growth restriction (FGR) was made antenatally on the basis of ultrasound diagnosis, which includes estimated fetal weight below 1.5 SD of the Japanese fetal weight standard by gestational age, serial changes in estimated fetal weight, and abdominal circumference^32^.

Each facility in the database was classified into the three categories of facility type: comprehensive perinatal centers, regional perinatal centers, and general maternity units^35^. Comprehensive perinatal centers are equipped with maternal-fetal and neonatal intensive care units and are responsible for receiving obstetric emergencies at all times. Regional perinatal centers are facilities that can also handle high-risk pregnancies, but would transfer women who are at risk of early preterm delivery. Other obstetric hospitals and clinics were considered general maternity units.

### Statistical analysis

Descriptive statistics of the study sample and the stillbirth rates per 1,000 births were calculated for each variable. The smoothed rate of stillbirth by gestational was also obtained using the local polynomial smoothing method. Poisson regression analyses were performed to examine the association between stillbirth and explanatory variables. A multi-level model with a facility-level random effect was used in order to adjust for the effect of clustering of patients with specific conditions within facilities and unmeasured variations between facilities that may be associated with stillbirth risk (e.g., number of physicians). The variables entered in the multi-level models were those that showed significance level of P-value ≤ 0.20 in the simple analyses (overall or within the subgroups)^27^. Backward-stepwise model building was manually performed for model selection^36^. Sensitivity analysis was conducted including subjects with missing or implausible data. An additional analysis was performed to examine the effect of interaction between SGA and antenatal-diagnosis of FGR, because SGA infants include those who are constitutionally normal but small due to maternal size or genetic deposition and those who are pathologically growth-restricted due to certain conditions (e.g., placental insufficiency or chromosomal abnormalities)^34,37^. P-value ≤ 0.05 were considered statistically significant. All analyses were performed using Stata/MP 15.0 (Stata corporation, College Station, TX).

### Ethical Approval

This study was approved by the Research Ethics Committee at the University of Tokyo (number: 11009) and the Clinical Research Management and Review Committee of the Japan Society of Obstetrics and Gynecology (number: 23). All methods were performed in accordance with the relevant guidelines and regulations of the institutions. Informed consent was obtained from patients for the use of their data collected during routine clinical practice for medical research purposes.

### Data Availability

The datasets generated during and/or analyzed during the current study are available upon request to the Japan Society of Obstetrics and Gynecology (nissanfu@jsog.or.jp). The data used in this study cannot be shared by the authors under the conditions imposed on data users by the society.

## Results

### Causes of singleton stillbirth

Table 2 presents the cause of death for 2,133 singleton stillbirths by the JSOG clinical death classification system and matched level 1 categories of CODAC system by gestational age groups. Among 2,133 stillbirths, 822 (38.5%) occurred in the extremely preterm period (i.e., 22–27 weeks of gestation), 403 (18.9%) in the very preterm period (i.e., 28–31 weeks), 522 (24.5%) in the moderate to late preterm period (i.e., 32–36 weeks), and 386 (18.1%) at term (i.e., over 37 weeks)^38^. Cause of death was unknown in 25–40% of stillbirths across gestational age. Most cases categorized as others did not have a relevant clinical diagnosis in the free-text column, so the details remained unknown. Placental abnormality contributed the largest proportion of stillbirths with a known cause (21.7%), followed by cord abnormality (15.6%) and congenital malformation (15.1%). Intrapartum, infection, maternal and fetal conditions accounted for less than 8%. Among stillbirths attributed to umbilical cord abnormality with more detailed pertinent conditions recorded, excessive coiling was identified as the cause in 53.2% followed by collapse or stricture (11.9%) and multiple loops around fetal body (10.2%).

**Table 2:** Causes of singleton stillbirth and proportions receiving autopsy and placental pathology examination.

CODAC system (Level 1)	JSOG clinical death classification system	Number of events (%)					Autopsy (%)	Placental pathology (%)
		22–27 wks.	28–31 wks.	32–36 wks.	37–45 wks.	Total		
Maternal	Pregnancy-induced hypertension	26 (3.2)	13 (3.2)	6 (1.1)	7 (1.8)	82 (3.8)	5 (6.1)	36 (43.9)
	Other maternal complication	14 (1.7)	6 (1.5)	7 (1.3)	3 (0.8)			
Placenta	Placenta previa	4 (0.5)	1 (0.2)	1 (0.2)	0 (0.0)	462 (21.7)	10 (2.2)	202 (43.7)
	Placental abruption	39 (4.7)	72 (17.9)	152 (29.1)	69 (17.9)			
	Other placenta abnormality	68 (8.3)	24 (6.0)	14 (2.7)	18 (4.7)			
Cord	Umbilical cord abnormality	134 (16.3)	65 (16.1)	73 (14.0)	61 (15.8)	333 (15.6)	15 (4.5)	157 (47.2)
Intrapartum	Abnormal fetal lie, attitude, or rotation	3 (0.4)	0 (0.0)	1 (0.2)	2 (0.5)	23 (1.1)	3 (12.5)	9 (37.5)
	Fetal hypoxia with other causes	8 (1.0)	2 (0.5)	3 (0.6)	4 (1.0)			
Fetal	Fetal trauma	1 (0.1)	0 (0.0)	0 (0.0)	0 (0.0)	168 (7.9)	12 (7.1)	65 (38.7)
	Fetal hemolytic disorder	3 (0.4)	0 (0.0)	1 (0.2)	0 (0.0)			
	Non-immune fetal hydrops	32 (3.9)	23 (5.7)	11 (2.1)	2 (0.5)			
	Low birth weight with other causes	67 (8.2)	21 (5.2)	5 (1.0)	2 (0.5)			
Congenital	Congenital malformation	92 (11.2)	61 (15.1)	107 (20.5)	61 (15.8)	321 (15.1)	26 (8.1)	87 (27.1)
Infection	Perinatal infection	33 (4.0)	3 (0.7)	3 (0.6)	5 (1.3)	44 (2.1)	2 (4.3)	25 (56.8)
Unknown	Others	286 (34.8)	110 (27.3)	135 (25.9)	144 (37.3)	700 (32.8)	28 (4.0)	276 (39.4)
	Miscoded	7 (0.9)	0 (0.0)	0 (0.0)	1 (0.3)			
	Missing	5 (0.6)	2 (0.5)	3 (0.6)	7 (1.8)			
Total		822	403	522	386	2,133 (100)	101 (4.7)	857 (40.2)

CODAC: Cause of Death and Associated Conditions, JSOG: Japan Society of Obstetrics and Gynecology, wks.: weeks of gestation.

The proportions of stillbirths receiving autopsy or placental pathology examination are also shown in Table 2. Overall, the autopsy rate was very low (4.7%), and even lower among cases with unknown cause of death (4.0%). Among 101 cases that underwent autopsy with recorded results, 70.3% showed clinical diagnoses that would have contributed to death. Placental pathology examination was performed for 40.2% of stillbirths. A wide variation in proportion of stillbirths with autopsy and placental pathology examination was observed regardless of facility type or average annual delivery volume (Supplementary Figure S1). Even among comprehensive perinatal centers, autopsy rates varied between 0–40% and placental examination rates between 0–100%.

### Characteristics of non-malformed singleton births

Table 3 shows the descriptive statistics of 270,450 non-malformed singleton births including 1,075 stillbirths by maternal, fetal, and facility characteristics. The mean maternal age was 32.2 years. More than half were nulliparous. The majority of women had a normal weight and 21.9% had BMI over 23 kg/m^2^. Active smoking was observed in 3.4% of women. Eighty percent of births in this dataset were collected at comprehensive or regional perinatal centers.

**Table 3:** Characteristics and crude relative risk of factors associated with non-malformed singleton stillbirth.

Risk factors	Total births	Stillbirths	Crude relative risk (95% CI)	P-value
	No. (%)	No. (Rate per 1000 births)		
Total	270,450	1,075		
Maternal age, years				
Mean	32.2 ± 5.4	32.3 ± 5.6		
<20	3,695 (1.4)	17 (4.6)	1.19 (0.74–1.93)	0.5
20–34	169,382 (62.6)	653 (3.9)	1.00	NA
≥35	97,373 (36.0)	405 (4.2)	1.08 (0.95–1.22)	0.2
Parity				
0	140,686 (52.0)	601 (4.3)	1.17 (1.04–1.32)	0.01
≥1	129,764 (48.0)	474 (3.7)	1.00	NA
Pre-pregnancy BMI, kg/m^2^				
Mean	21.3 ± 3.6	21.7 ± 4.0		
<18.5	47,091 (17.4)	171 (3.6)	0.95 (0.80–1.12)	0.6
18.5–22.9	164,004 (60.6)	627 (3.8)	1.00	NA
23.0–29.9	50,316 (18.6)	228 (4.5)	1.19 (1.02–1.38)	0.03
≥30.0	9,039 (3.3)	49 (5.4)	1.42 (1.06–1.90)	0.02
Smoking				
No	261,163 (96.6)	1,016 (3.9)	1.00	NA
Yes	9,287 (3.4)	59 (6.4)	1.63 (1.26–2.12)	<0.001
Use of ART				
No	251,782 (93.1)	994 (3.9)	1.00	NA
Yes	18,668 (6.9)	81 (4.3)	1.10 (0.88–1.38)	0.4
Pre-existing hypertension				
No	268,072 (99.1)	1,040 (3.9)	1.00	NA
Yes	2,378 (0.9)	35 (14.7)	3.79 (2.71–5.31)	<0.001
Pre-existing diabetes mellitus				
No	268,357 (99.2)	1,064 (4.0)	1.00	NA
Yes	2,093 (0.8)	11 (5.3)	1.33 (0.73–2.40)	0.4
Thyroid disease				
No	262,029 (96.9)	1,046 (4.0)	1.00	NA
Yes	8,421 (3.1)	29 (3.4)	0.86 (0.60–1.25)	0.4
History of stillbirth^a^				
No	127,372 (98.2)	456 (3.6)	1.00	NA
Yes	2,392 (1.8)	18 (7.5)	2.10 (1.31–3.37)	0.002
History of preterm birth^a^				
No	122,382 (94.3)	435 (3.6)	1.00	NA
Yes	7,382 (5.7)	39 (5.3)	1.49 (1.07–2.06)	0.02
History of cesarean section^a^				
No	103,408 (79.7)	389 (3.8)	1.00	NA
Yes	26,356 (20.3)	85 (3.2)	0.86 (0.68–1.08)	0.2
PIH				
No	255,677 (94.5)	978 (3.8)	1.00	NA
Yes	14,773 (5.5)	97 (6.6)	1.72 (1.39–2.11)	<0.001
Amniotic fluid volume				
Oligohydramnios	4,858 (1.8)	68 (14.0)	3.73 (2.92–4.77)	<0.001
Normal	264,291 (97.7)	991 (3.7)	1.00	NA
Polyhydramnios	1,301 (0.5)	16 (12.3)	3.28 (2.00–5.37)	<0.001
Infant sex				
Male	139,116 (51.4)	546 (3.9)	1.00	NA
Female	131,334 (48.6)	529 (4.0)	1.03 (0.91–1.16)	0.7
Infant size				
SGA	26,991 (10.0)	486 (18.0)	7.25 (6.41–8.20)	<0.001
AGA	215,364 (79.6)	535 (2.5)	1.00	NA
LGA	28,095 (10.4)	54 (1.9)	0.77 (0.58–1.02)	0.07
Facility type				
Comprehensive perinatal center	90,190 (33.4)	502 (5.6)	1.00	NA
Regional perinatal center	125,320 (46.3)	448 (3.6)	0.64 (0.57–0.73)	<0.001
General maternity unit	54,9401 (20.3)	125 (2.3)	0.41 (0.34–0.50)	<0.001

^a^Analyses restricted to multiparous women (Births: 129,764; Stillbirths: 474).

ART: assisted reproductive technology, AGA: appropriate-for-gestational-age, BMI: body mass index, CI: confidence interval, LGA: large-for-gestational-age, NA: not applicable, PIH: pregnancy-induced hypertension, SGA: small-for-gestational-age.

### Risk factors for non-malformed singleton stillbirth

**Figure 2:**
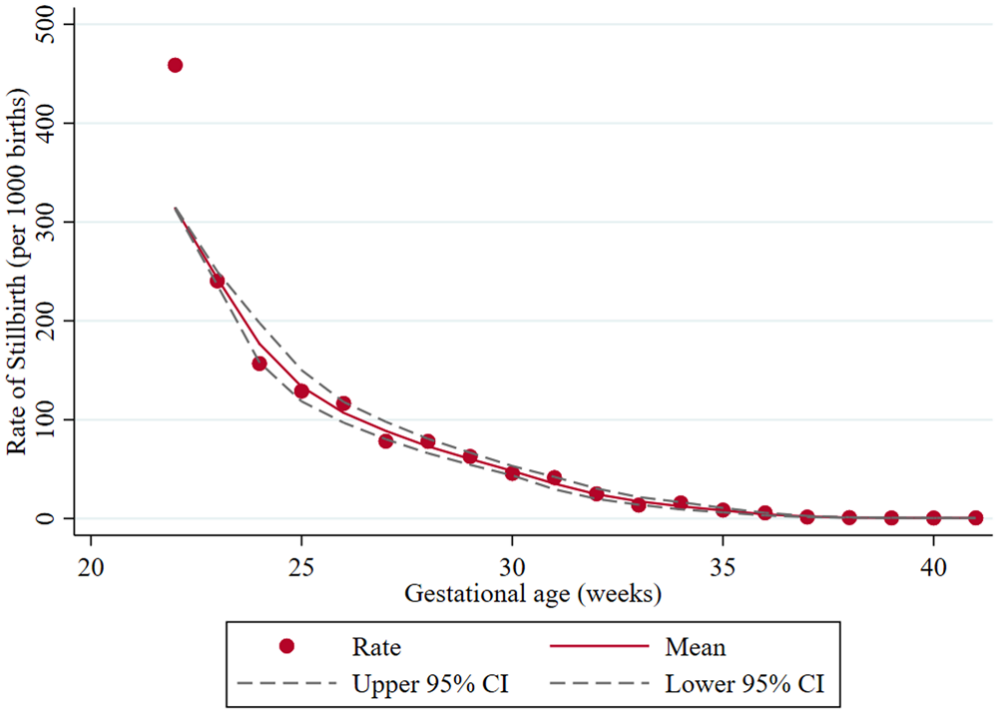
Smoothed rates of stillbirth by gestational age. The smoothed curve of stillbirth rates by gestational age is shown. The rate was highest at 22 weeks (458.8 per 1000 births) and lowest at 40 weeks of gestation (0.5 per 1000 births).

Figure 2 presents the smoothed curve of stillbirth rates by gestational age. The rate was highest at 22 weeks (458.8 per 1000 births) and lowest at 40 weeks of gestation (0.5 per 1000 births). Crude relative risks of factors associated with stillbirth are also shown in Table 3. Women who were nulliparous, overweight or obese, or smoked, were more likely to experience stillbirth. Stillbirths were also more frequently seen in women with pre-existing hypertension, history of stillbirth, history of preterm birth, PIH, and abnormal amniotic fluid volume. SGA infants had a much higher risk of stillbirth compared to infants of normal size. Comprehensive perinatal centers were two times more likely to report stillbirths than general maternal units.

Table 4 shows the result of the multi-level Poisson regression analysis. Nulliparous women continued to have a significantly higher risk compared to multiparous women (adjusted relative risk [ARR]: 1.19, 95% confidence interval [CI]: 1.05–1.35, P = 0.006). Maternal underweight showed a protective effect (ARR: 0.82, 95% CI: 0.69–0.97, P = 0.02), while overweight and obesity no longer showed an increased risk of stillbirth. Interestingly, PIH (ARR: 0.31, 95% CI: 0.25–0.39, P < 0.001) and oligohydramnios (ARR: 0.66, 95% CI: 0.51–0.86, P = 0.002) were associated with a reduced risk. The risk related with SGA was attenuated but remained significant (ARR: 3.78, 95% CI: 3.31–4.32, P < 0.001). The result of the sensitivity analysis showed a similar pattern in the risk of stillbirth (see Supplementary Table S3). A separate analysis including the interaction between SGA and antenatally-diagnosed FGR showed that while SGA infants with FGR had twice the risk of stillbirth compared to non-SGA non-FGR infants (ARR: 1.92, 95% CI: 1.54–2.40, P < 0.001), those without had five times the risk (ARR: 4.86, 95% CI: 4.23–5.58, P < 0.001) (see Supplementary Table S4).

**Table 4:** Adjusted relative risk of non-malformed singleton stillbirth.

Risk factors	Adjusted relative risk^*^	95% CI	P-value
Maternal age, years			
<20	0.94	0.58–1.52	0.8
20–34	1.00	NA	NA
≥35	1.05	0.92–1.19	0.5
Parity			
0	1.19	1.05–1.35	0.006
≥1	1.00	NA	NA
Pre-pregnancy BMI, kg/m^2^			
<18.5	0.82	0.69–0.97	0.02
18.5–22.9	1.00	NA	NA
23.0–29.9	1.04	0.89–1.21	0.6
≥30.0	1.12	0.83–1.50	0.5
Smoking			
No	1.00	NA	NA
Yes	1.13	0.86–1.47	0.4
PIH			
No	1.00	NA	NA
Yes	0.31	0.25–0.39	<0.001
Amniotic fluid volume			
Oligohydramnios	0.66	0.51–0.86	0.002
Normal	1.00	NA	NA
Polyhydramnios	1.39	0.84–2.29	0.2
Infant size			
SGA	3.78	3.31–4.32	<0.001
AGA	1.00	NA	NA
LGA	0.97	0.73–1.28	0.8

^*^Adjusted for gestational age and explanatory variables in the table.

AGA: appropriate-for-gestational-age, BMI: body mass index, CI: confidence interval, LGA: large-for-gestational-age, NA: not applicable, PIH: pregnancy-induced hypertension, SGA: small-for-gestational-age.

## Discussion

This study examined singleton stillbirth in Japan using a nationwide perinatal database between 2013 and 2014. To our knowledge, this is the first study to examine cause of death by gestational age and identify key risk factors for stillbirth in the country. Our study found that the cause of death remained unknown in about one third of stillbirths with low rates of autopsy and placental examination. We also found that while small-sized infants and nulliparous women had a significantly increased risk of stillbirth, other well-known clinical risk factors for perinatal mortality, such as maternal overweight/obesity, PIH and oligohydramnios, had no or even protective effect on stillbirth risk.

The JSOG Perinatal Database uses a unique JSOG clinical death classification system for recording cause of perinatal death. Although there are methodological difficulties in matching the results of different classification systems^3,39^, we reclassified the cause of death using the CODAC system in order to compare the results with those from other countries. In our analysis, consistent with analyses in other developed nations, placental abnormality was identified as the cause of death in one fifth of stillbirths, followed by cord abnormality and congenital malformation. Infection, maternal, and fetal conditions accounted for only a small proportion. Flenady and colleagues analyzed 617 stillbirths of 22 weeks of gestation or 500 g or greater birth weight in six high-income western countries^2^. They found that placental abnormality accounted for 29% of stillbirths, followed by infection 12%, cord abnormality 9%, maternal cause 7%, congenital anomaly 6%, fetal cause 4%, and intrapartum event 3%. Cause of death was classified as unknown in 30% of stillbirths. Helgadottir and colleagues, who analyzed 377 stillbirths after 22 completed weeks of gestation in Norway, showed that placental abnormality accounted for about half (50.4%) of all deaths and infection 12.2%, while other causes contributed less than 10%^40^. Cause of death was unknown in 19.4% of stillbirths. The largest contribution of placental abnormality as the cause of stillbirth is further supported by a systematic review of 41 studies that examined placental pathology findings in association with stillbirth^41^. However, the proportions of congenital malformation and umbilical abnormality in our study were much higher compared to previous studies^2,40,42^. This high proportion of congenital malformation is probably because the cases registered in the database are biased towards high-risk pregnancy, due to the nature of the facilities registered. Umbilical cord abnormalities such as excessive coiling and loops are often seen in live births. Therefore, aside from clinically obvious or pathologically diagnosed cases, it is difficult to attribute umbilical cord abnormality as a direct cause of death and the proportion presented in this study may be overestimated^2,42,43^.

Identifying a single direct cause of stillbirth is difficult in many cases and uncertainty may exist even after a full investigation^43^. Nevertheless, investigating why and how it occurred is an important process for both physicians and families experiencing the trauma of stillbirth. It may reveal conditions that may predispose families to recurrent stillbirth and help better prepare for future pregnancies, and assist families to come to terms with lost pregnancies^3,43,44^. It may also provide important information to develop strategies to further prevent stillbirth at each facility and improve the local perinatal system.

Ideally, postmortem investigation should include comprehensive maternal history, maternal laboratory tests for common etiologies, clinical examination and autopsy of the infant, and macroscopic and pathological examination of the placenta and umbilical cord^2,3,43^. The proportion of stillbirths with unknown cause in our study was comparable with other studies^2,42^, but the rates of autopsy and placental examination were significantly lower, although these tests are recommended in the JSOG guideline when the cause of death is unclear^32^. Whether or not the tests are implemented may depend on the availability of pathology service and specialized staff^3,43^. However, a wide variation in the proportion of stillbirths receiving the tests, regardless of facility type or delivery volume, suggests that some facilities are more suitable or proactive in offering and carrying out the tests than others. Since a physician’s view on the tests can impact family’s decision making, physicians must correctly understand the value and limitations of each test^3,43,44^. Offering less invasive postpartum investigation using magnetic resonance imaging or small-incision tissue biopsy is another option^32,43,45^. However, since facilities that can provide them are still limited^46^, a practical option may be to establish a network of hospitals that can offer the services as is under way in the United Kingdom^45^. In all cases, families should be offered the choice of postmortem investigation and have the right to understand about their loss.

In addition, because multiple conditions may contribute to stillbirth, it is recommended to record the chain of events that led to death, rather than the single most probable cause of death^18,39,43^. The recently developed coding system, the WHO application of ICD-10 to deaths during the perinatal period (ICD-PM), requires entering of all contributing maternal and fetal conditions in addition to the timing of perinatal death (antepartum, intrapartum, or neonatal)^47^. In our study, the ICD-PM system could not be used to examine causes of stillbirth because of lack of this information. The ICD-PM is intended to standardize the recording of perinatal deaths and enhance comparison of data internationally. Implementation of this coding system in the JSOG Perinatal Database may enable us to identify conditions or mechanisms that are currently not identified, which may explain some of the stillbirths with unknown cause of death, and its implementation should be hastened in Japan.

In the risk factor analysis, advanced maternal age was not statistically significant, although it has been known to be one of the major risk factors for stillbirth^10,20,26^. The increase in stillbirth risk with advanced age is often attributed to the combined effect of uteroplacental insufficiency, influence of chronic and obstetric complications, and higher risk of genetic abnormalities in infants^20,48^. The reason for no significant association in this study may be because we adjusted for a number of known complications, excluded infants with congenital malformation, and women in advanced age are closely followed-up in tertiary or secondary facilities, averting the risk.

Nulliparous women had a small but significant increase in stillbirth risk, consistent with previous studies^10,20,26,27^. The biologic mechanism explaining the increased risk in nulliparous women is not well-documented, but one hypothesis is that they have higher vascular resistance and lower blood flow in the uteroplacental arteries compared to multiparous women^49^.

Overweight and obesity did not have a significant association with stillbirth, but underweight showed a protective effect. A systematic review that examined a dose-response relationship between pre-pregnancy BMI and stillbirth risk found an almost linear curve, with underweight women having a reduced risk compared to women with a BMI of 20 kg/m^2^ ^19^. While maternal underweight is associated with increased risk of antenatal anemia, preterm delivery, and low birth weight, the protective effect on stillbirth may be explained by lower prevalence of other complications such as pre-eclampsia and interventions during labor^50,51^.

Maternal complications and obstetric history showed no increase in the risk of stillbirth. PIH and oligohydramnios even showed a reduced risk after adjusting for confounders. A similar finding was observed in a study from England, in which pre-eclampsia was associated with a reduced risk of stillbirth between 24 and 33 weeks of gestation (ARR: 0.3, 95% CI: 0.1–0.6)^20^. This is probably because infants are delivered early due to maternal reasons, which reduces the stillbirth risk before the disease becomes symptomatic^20^. This would apply to other relevant conditions, as these women are considered high-risk and followed up especially closely. This finding supports that better management of maternal conditions can reduce stillbirth in countries with higher stillbirth rates but functional obstetric care frameworks.

SGA infants had a significant increase in risk, consistent with previous studies. About 30% of SGA infants had an antenatal diagnosis of FGR, but the remaining 70% did not. The analysis of the interaction between SGA and FGR showed that SGA infants without antenatal diagnosis of FGR had a much higher risk of stillbirth compared with those with the diagnosis. This is comparable with the study by Gardosi and colleagues, who reported a reduced risk when FGR was detected antenatally (ARR: 3.4, 95% CI: 2.2–5.2) to when it was not (ARR: 6.5, 95% CI: 4.9–8.4)^20^. Clinical FGR is a well-established risk factor for perinatal mortality. Once it is detected, the fetus is carefully monitored and often delivered before severe deterioration is observed on Doppler ultrasound or cardiotocography^37^. On the other hand, non-malformed fetuses that are small but do not meet the diagnosis of FGR are considered to have a relatively lower risk of death and their early delivery may be delayed. Although the timing of delivery requires careful consideration of various competing risks, given the potential preventability of stillbirth with antenatal detection of FGR, small-sized fetuses should be more carefully monitored and treated as having stillbirth risk. It should also be noted that fetal weight loss may occur as a result of antepartum stillbirth^52^, therefore the effect of SGA (defined using birth weight) on stillbirth could be overestimated.

This study utilized a nationwide perinatal database that contains a wide range of clinical information, which is not available from other existing databases such as the vital registration system. Also, by using a multi-level model, we were able to adjust for the effect of clustering and unmeasured facility-specific factors. However, despite these strengths, several limitations should be noted. First, the results of this study may not be generalizable to the whole population, because the cases registered in this database are mostly from secondary and tertiary facilities and the stillbirth rate was much higher than from the vital statistics. To maximize the benefit of the JSOG Perinatal Database and enable nationally representative analyses, more participation of general maternal units in the registry is needed. Nevertheless, the findings in this study will contribute to clinical knowledge of stillbirth among high-risk pregnancies. Second, 27.0% of the women were excluded from the risk factor analysis because of missing or implausible data, mainly on pre-pregnancy weight, height, and smoking status. The sensitivity analysis that considered missing data in the regression models produced similar results, indicating that selection bias caused by missing data is not a major concern. Even so, improved data collection and recording of maternal baseline characteristics is needed in order to improve future management of women at risk of stillbirth. Third, although different approaches will be required for prevention of stillbirth before and after the onset of labor, fresh and macerated stillbirths could not be differentiated due to the lack of data. Similarly, socioeconomic factors such as household income, education, occupation, and number of antenatal care visits were not available. These factors are well-known risk factors for stillbirth as well as major determinants of disparity in access to perinatal care^3,10,42^. Despite the provision of public financial support to all pregnant women, a report showed that about 0.3% do not receive antenatal care mainly due to financial difficulties^53^. Better recording of these risk factors would be crucial for future epidemiological studies on perinatal health in Japan.

## Conclusion

Our study suggests that stillbirths occurring among women with known complications are likely being prevented, at least in secondary and tertiary facilities. Further reduction in stillbirths must target small-sized fetuses and nulliparous women. Improvement in postpartum investigation and recording of the causal pathways of stillbirths may enable us to explain some of the stillbirths with unknown cause of death, and to continue to make progress in reducing this tragic pregnancy complication.

## Electronic supplementary material

Supplementary Information

## References

[1] Blencowe H (2016). National, regional, and worldwide estimates of stillbirth rates in 2015, with trends from 2000: a systematic analysis. Lancet Glob Health.

[2] Flenady V (2011). Stillbirths: the way forward in high-income countries. Lancet..

[3] Flenady V (2016). Stillbirths: recall to action in high-income countries. Lancet..

[4] Maeda K (2013). Progress of Perinatal Medicine in Japan. J Health Med Informats.

[5] Maeda K (2014). Highly improved perinatal states in Japan. J Obstet Gynaecol Res.

[6] Ministry of Health Labor and Welfare Japan. *Vital Statistics*, http://www.mhlw.go.jp/english/database/db-hw/index.html (2015).

[7] Sugai MK, Gilmour S, Ota E, Shibuya K (2017). Trends in perinatal mortality and its risk factors in Japan: Analysis of vital registration data, 1979-2010. Sci Rep..

[8] Yoneda M, Yoshida K, Soyama S, Shimada K (2015). Post-discharge perinatal grief care and tentative design of a regional cooperation system. J Tsuruma Health Sci Soc.

[9] Sato S (2007). Epidemiology of stillbirth in Japan using The Japan Society of Obstetrics and Gynecology PerinatalDatabase (in Japanese). J Jpn Soc Perinat Neonat Med.

[10] Flenady V (2011). Major risk factors for stillbirth in high-income countries: a systematic review and meta-analysis. Lancet..

[11] NCD Risk (2016). Factor Collaboration. Trends in adult body-mass index in 200 countries from 1975 to 2014: a pooled analysis of 1698 population-based measurement studies with 19.2 million participants. Lancet..

[12] Hasegawa J (2016). Relevant Obstetric Factors for Cerebral Palsy: From the Nationwide Obstetric Compensation System in Japan. PLoS One.

[13] Hayashi M, Nakai A, Satoh S, Matsuda Y (2012). Adverse obstetric and perinatal outcomes of singleton pregnancies may be related to maternal factors associated with infertility rather than the type of assisted reproductive technology procedure used. Fertil Steril.

[14] The Japan Society of Obstetrics and Gynecology. Perinatal Database Report (2015). 2013 (in Japanese). Acta Obstet Gynaecol Jpn.

[15] The Japan Society of Obstetrics and Gynecology. *The Japan Society of Obstetrics and Gynecology Perinatal Committee Reports* (in Japanese), http://www.jsog.or.jp/activity/report.html (2017).

[16] Ministry of Health Labor and Welfare Japan. *Survey of Medical Institutions*, http://www.mhlw.go.jp/english/database/db-hss/smi.html (2014).

[17] Japan Association of Obstetricians and Gynecologists. *Definition of Induced Abortion* (in Japanese), http://www.jaog.or.jp/sep2012/JAPANESE/teigen/teigi.htm (2012).

[18] Froen JF (2009). Causes of death and associated conditions (Codac): a utilitarian approach to the classification of perinatal deaths. BMC Pregnancy Childbirth.

[19] Aune D, Saugstad OD, Henriksen T, Tonstad S (2014). Maternal body mass index and the risk of fetal death, stillbirth, and infant death: a systematic review and meta-analysis. JAMA.

[20] Gardosi J, Madurasinghe V, Williams M, Malik A, Francis A (2013). Maternal and fetal risk factors for stillbirth: population based study. BMJ.

[21] Haws RA (2009). Reducing stillbirths: screening and monitoring during pregnancy and labour. BMC Pregnancy Childbirth.

[22] Lamont K, Scott NW, Jones GT, Bhattacharya S (2015). Risk of recurrent stillbirth: systematic review and meta-analysis. BMJ.

[23] Marufu TC, Ahankari A, Coleman T, Lewis S (2015). Maternal smoking and the risk of still birth: systematic review and meta-analysis. BMC Public Health.

[24] Mondal D, Galloway TS, Bailey TC, Mathews F (2014). Elevated risk of stillbirth in males: systematic review and meta-analysis of more than 30 million births. BMC Med.

[25] O’Neill SM (2014). Cesarean section and rate of subsequent stillbirth, miscarriage, and ectopic pregnancy: a Danish register-based cohort study. PLoS Med.

[26] Reddy UM (2010). Prepregnancy risk factors for antepartum stillbirth in the United States. Obstet Gynecol.

[27] Stillbirth Collaborative Research Network Writing Group (2011). Association between stillbirth and risk factors known at pregnancy confirmation. JAMA.

[28] Trudell AS, Cahill AG, Tuuli MG, Macones GA, Odibo AO (2013). Risk of stillbirth after 37 weeks in pregnancies complicated by small-for-gestational-age fetuses. Am J Obstet Gynecol.

[29] Surkan PJ, Stephansson O, Dickman PW, Cnattingius S (2004). Previous preterm and small-for-gestational-age births and the subsequent risk of stillbirth. N Engl J Med.

[30] Marlow N (2014). Perinatal outcomes for extremely preterm babies in relation to place of birth in England: the EPICure 2 study. Arch Dis Child Fetal Neonatal Ed.

[31] Snowden JM, Cheng YW, Emeis CL, Caughey AB (2015). The impact of hospital obstetric volume on maternal outcomes in term, non-low-birthweight pregnancies. Am J Obstet Gynecol.

[32] Minakami H (2014). Guidelines for obstetrical practice in Japan: Japan Society of Obstetrics and Gynecology (JSOG) and Japan Association of Obstetricians and Gynecologists (JAOG) 2014 edition. J Obstet Gynaecol Res.

[33] WHO expert consultation (2004). Appropriate body-mass index for Asian populations and its implications for policy and intervention strategies. Lancet..

[34] Itabashi K, Miura F, Uehara R, Nakamura Y (2014). New Japanese neonatal anthropometric charts for gestational age at birth. Pediatr Int.

[35] Ministry of Health Labor and Welfare Japan. *Current Perinatal Care System of Japan* (in Japanese), http://www.mhlw.go.jp/file/05-Shingikai-10801000-Iseikyoku-Soumuka/0000096037.pdf (2015).

[36] Armitage, P., G., B. & Matthews, J. N. S. *Statistical methods in medical research*. 4 edn, (John Wiley & Sons, 2013).

[37] Alberry M, Soothill P (2007). Management of fetal growth restriction. Arch Dis Child Fetal Neonatal Ed.

[38] World Health Organization. *Preterm birth*. http://www.who.int/mediacentre/factsheets/fs363/en/ (2017).

[39] Flenady V (2009). An evaluation of classification systems for stillbirth. BMC Pregnancy Childbirth.

[40] Helgadottir LB (2013). Classification of stillbirths and risk factors by cause of death–a case-control study. Acta Obstet Gynecol Scand.

[41] Ptacek I, Sebire NJ, Man JA, Brownbill P, Heazell AE (2014). Systematic review of placental pathology reported in association with stillbirth. Placenta.

[42] Stillbirth Collaborative Research Network Writing Group (2011). Causes of death among stillbirths. JAMA.

[43] Silver RM (2007). Work-up of stillbirth: a review of the evidence. Am J Obstet Gynecol.

[44] Faye-Petersen OM, Guinn DA, Wenstrom KD (1999). Value of perinatal autopsy. Obstet Gynecol.

[45] Arthurs OJ, Bevan C, Sebire NJ (2015). Less invasive investigation of perinatal death. BMJ.

[46] Okuda T, Shiotani S, Sakamoto N, Kobayashi T (2013). Background and current status of postmortem imaging in Japan: short history of “Autopsy imaging (Ai)”. Forensic Sci Int.

[47] World Health Organization. The WHO application of ICD-10 to deaths during the perinatal period: ICD-PM. WHO Library Cataloguing-in-Publication Data (2016).

[48] Fretts R, Schmittdiel J, McLean F, Usher R, Goldman M (1995). Increased maternal age and the risk of fetal death. N Engl J Med.

[49] Waldenstrom U, Cnattingius S, Norman M, Schytt E (2015). Advanced Maternal Age and Stillbirth Risk in Nulliparous and Parous Women. Obstet Gynecol.

[50] Rahman MM (2015). Maternal body mass index and risk of birth and maternal health outcomes in low- and middle-income countries: a systematic review and meta-analysis. Obes Rev.

[51] Sebire NJ, Jolly M, Harris J, Regan L, Robinson S (2001). Is maternal underweight really a risk factor for adverse pregnancy outcome? A population-based study in London. BJOG.

[52] Chard T (2001). Does the fetus lose weight in utero following fetal death: a study in preterm infants. BJOG..

[53] Unno N (2011). The Perinatal Care System in Japan. JMAJ.

