# Supplementary material for: Causes and risk factors for singleton stillbirth in Japan: Analysis of a nationwide perinatal database, 2013–2014
Source: Sci Rep. 2018 Mar 7;8:4117. doi: 10.1038/s41598-018-22546-9 (PMC5841302; doi:10.1038/s41598-018-22546-9)

Supplementary Information

Causes and risk factors for singleton stillbirth in Japan: Analysis of a nationwide perinatal database, 2013-2014

Rei Haruyama, Stuart Gilmour, Erika Ota, Sarah K. Abe, Md. Mizanur Rahman, Shuhei Nomura, Naoyuki Miyasaka, and Kenji Shibuya

Supplementary Table S1: Comparison of characteristics between included and excluded subjects

In the risk factor analysis, subjects with missing or implausible data were excluded. The excluded women were more likely to deliver in the preterm period, slightly younger, more often overweight or obese and smokers.

	Included subjects No. (%)	Excluded subjects No. (%)	P-value (Chi-squared test)
Gestational age, weeks			
22-27	2,747 (1.0)	1,494 (1.5)	<0.001
28-31	3,798 (1.4)	1,998 (2.0)	
32-36	23,378 (8.6)	10,847 (10.6)	
37-45	240,527 (88.9)	87,938 (86.0)	
Maternal age, years			
<20	3,695 (1.4)	1,502 (1.5)	<0.001
20-34	169,382 (62.6)	63,881 (63.1)	
≥35	97,373 (36.0)	35,789 (35.4)	
Parity			
0	140,686 (52.0)	51,973 (51.6)	0.01
≥1	129,764 (48.0)	48,855 (48.5)	
Pre-pregnancy BMI, kg/m^2			
<18.5	47,091 (17.4)	8,514 (17.2)	<0.001
18.5-22.9	164,004 (60.6)	29,323 (59.2)	
23.0-29.9	50,316 (18.6)	9,881 (20.0)	
≥30.0	9,039 (3.3)	1,799 (3.6)	
Smoking			
No	261,163 (96.6)	23,429 (95.9)	<0.001
Yes	9,287 (3.4)	999 (4.1)	

BMI: body mass index

Supplementary Table S2: Categories of the Japan Society of Obstetrics and Gynecology clinical death classification system

In the JSOG Perinatal Database, the single most probable cause of perinatal mortality is recorded using 17 categories. In our study, stillbirths attributed to “low birth weight with other causes” were classified as deaths due to unspecified fetal condition. Stillbirths attributed to “neonatal respiratory distress or hyaline membrane disease” and “twin-to-twin transfusion syndrome” were considered miscoded.

**Causes of perinatal death**
Pregnancy-induced hypertension
Other maternal complication
Placenta previa
Placental abruption
Other placental abnormality
Umbilical cord abnormality
Abnormal fetal lie, attitude, or rotation
Neonatal respiratory distress or hyaline membrane disease
Fetal/neonatal hypoxia with other causes
Fetal/neonatal trauma
Low birth weight with other causes
Congenital malformation
Fetal/neonatal hemolytic disorder
Perinatal infection
Twin-to-twin transfusion syndrome
Non-immune fetal hydrops
Others

Supplementary Table S3: Result of the sensitivity analysis

Sensitivity analysis was conducted including subjects with missing or implausible data in the regression models. It produced a similar result to the main analysis.

Risk factors	Adjusted relative risk*	95% CI	P-value
Maternal age, years			
<20	0.96	0.60-1.54	0.9
20-34	1.00	NA	NA
≥35	1.04	0.92-1.18	0.5
Parity			
0	1.20	1.06-1.35	0.004
≥1	1.00	NA	NA
Pre-pregnancy BMI, kg/m^2			
<18.5	0.82	0.69-0.97	0.02
18.5-22.9	1.00	NA	NA
23.0-29.9	1.03	0.89-1.20	0.7
≥30.0	1.10	0.82-1.47	0.5
Smoking			
No	1.00	NA	NA
Yes	1.13	0.87-1.47	0.4
PIH			
No	1.00	NA	NA
Yes	0.32	0.26-0.40	<0.001
Amniotic fluid volume			
Oligohydramnios	0.68	0.53-0.87	0.002
Normal	1.00	NA	NA
Polyhydramnios	1.38	0.84-2.28	0.2
Infant size			
SGA	3.58	3.14-4.08	<0.001
AGA	1.00	NA	NA
LGA	0.94	0.71-1.24	0.6

*Adjusted for gestational age and explanatory variables in the table

AGA: appropriate-for-gestational-age, BMI: body mass index, CI: confidence interval, LGA: large-for-gestational-age, NA: not applicable, PIH: pregnancy-induced hypertension, SGA: small-for-gestational-age

Supplementary Table S4: Interaction between small-for-gestational-age and fetal growth restriction

An additional analysis was performed to examine the effect of interaction between SGA and antenatal diagnosis of FGR. The result shows that while SGA infants with FGR had twice the risk of stillbirth compared to non-SGA non-FGR infants, those without had five times the risk.

	Total births No. (%)	Stillbirths No. (Rate per 1000 births)	Adjusted relative risk*	95% CI	P-value
SGA (−), FGR (−)	240,858 (89.1)	583 (2.4)	1.00	NA	NA
SGA (−), FGR (+)	2,601 (1.0)	6 (2.3)	0.66	0.29-1.48	0.3
SGA (+), FGR (−)	19,273 (7.1)	380 (19.7)	4.86	4.23-5.58	<0.001
SGA (+), FGR (+)	7,718 (2.9)	106 (13.7)	1.92	1.54-2.40	<0.001

*Adjusted for gestational age, maternal age, parity, pre-pregnancy BMI, smoking, maternal pregnancy-induced hypertension, and amniotic fluid volume

CI: confidence interval, FGR: fetal growth restriction, NA: not applicable, SGA: small-for-gestational-age

Supplementary Figure S1: Proportion of stillbirths with postmortem examinations by delivery volume and facility type

Proportions of stillbirths that underwent autopsy (red dot) and placental pathology examination (blue cross) by annual delivery volume of facilities are presented in each figure. Figure **a** shows the variation among comprehensive perinatal centers, **b** among regional perinatal centers, and **c** among general maternal units.

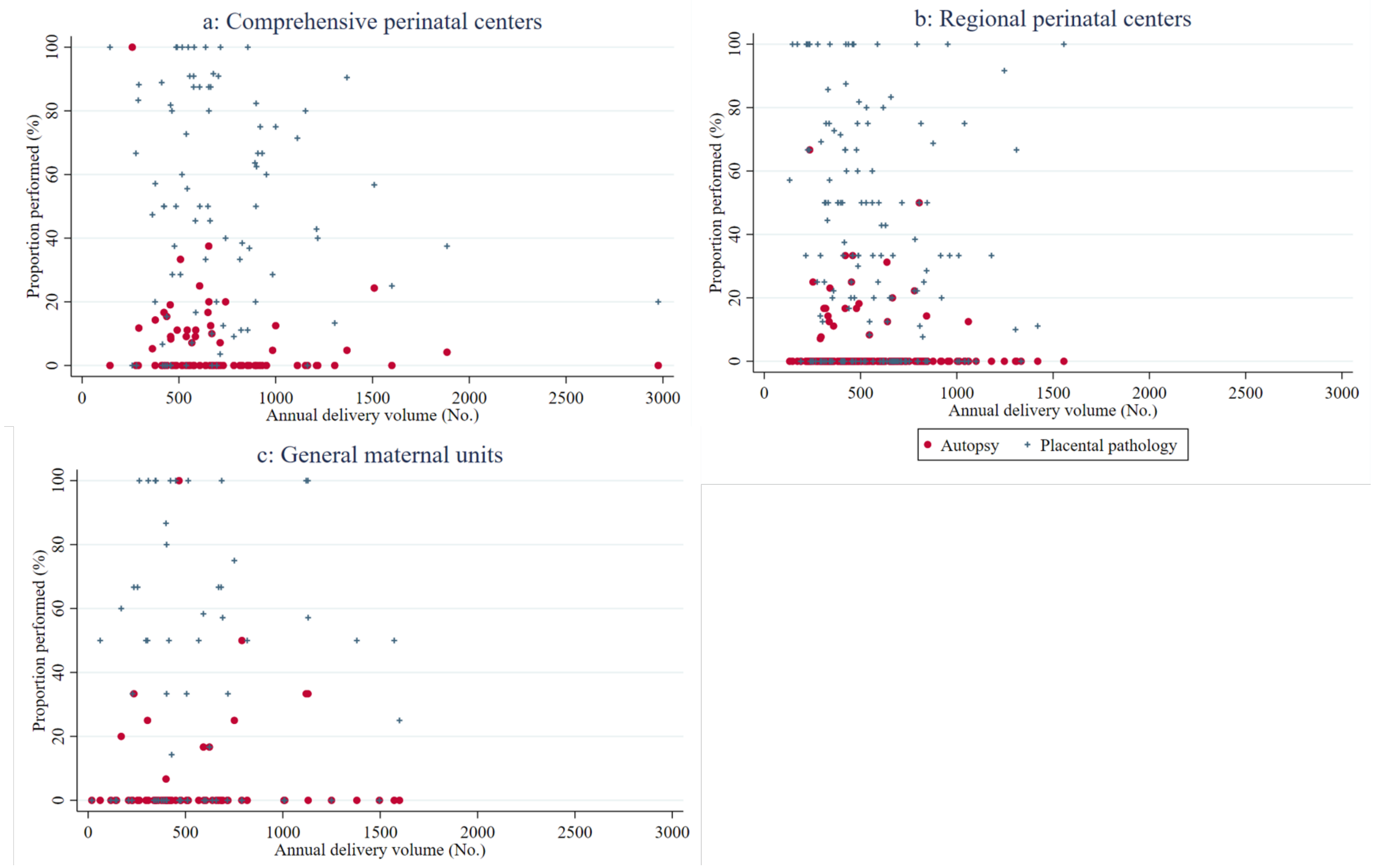

Supplement: Supplementary file 1 — Supplementary Information [file 41598_2018_22546_MOESM1_ESM.pdf]
